# Association between IGF-1 polymorphisms and risk of osteoporosis in Chinese population: a meta-analysis

**DOI:** 10.1186/s12891-018-2066-y

**Published:** 2018-05-10

**Authors:** Shu-tao Gao, Zheng-tao Lv, Chuan-kun Zhou, Chao Mao, Wei-bin Sheng

**Affiliations:** 1 Department of Spine Surgery, The First Affiliated Hospital of Xinjiang Medical University, Xinjiang, 830054 China; 2 Department of Orthopedics, Tongji Hospital, Tongji Medical College, Huazhong University of Science and Technology, Wuhan, 430030 Hubei China; 3 Department of Orthopedics, General Hospital of the Yangtze River Shipping, Wuhan, 430010 China

**Keywords:** IGF-1, Single nucleotide polymorphism, Osteoporosis, Meta-analysis

## Abstract

**Background:**

Several studies looking into the association between insulin-like growth factor-1 (IGF-1) gene polymorphisms and osteoporosis predisposition have been conducted among Chinese population with conflicting outcomes. The present systematic review and meta-analysis was performed to appraise and synthesize the existing evidence, so as to provide a more precise and reliable association between polymorphisms in IGF-1 gene and osteoporosis.

**Methods:**

Five electronic databases including PubMed, EMBASE, ISI Web of Science, CNKI and Wanfang were systematically searched for potential studies. Summary odds ratio (OR) and corresponding 95% confidence interval (95% CI) were calculated to evaluate the association. The best-matching genetic model of inheritance was determined using a genetic-model free approach.

**Results:**

Six case-control studies comprising 2068 osteoporosis patients and 2071 healthy controls were obtained for the meta-analysis. Dominant model was confirmed to be the best-matching genetic model (TT + TC versus CC). The overall data suggested that rs35767 polymorphism was significantly associated with osteoporosis vulnerability (OR 1.21, 95% CI 1.07, 1.37; *P* = 0.002). When stratifying the participants and performing subgroup-analysis according to source of patients, the result suggested that rs35767 was significantly correlated to osteoporosis in post-menopausal women subgroup (OR 1.29, 95% CI 1.08, 1.54; *P* = 0.005), but the correlation was not established in the subgroup of both gender (OR 1.14, 95% CI 0.96, 1.35; *P* = 0.12).

**Conclusion:**

Taken together, the findings of our current study suggested a significant association between rs35767 polymorphism and risk of osteoporosis in Chinese post-menopausal women.

**Electronic supplementary material:**

The online version of this article (10.1186/s12891-018-2066-y) contains supplementary material, which is available to authorized users.

## Background

Osteoporosis is a common disorder predominantly characterized by low bone mineral density (BMD) and micro-architectural deterioration of bone, which increases the bone fragility and leads to fracture. With the prolongation of human lifespan, more and more elderly people are suffering from osteoporosis. Osteoporosis has become a worldwide public-health problem [1, 2].

Similar to other complex chronic disease in the elderly, the precise etiopathogenesis underlying osteoporosis is also multifactorial. Risk factors such as genetics, age, physical activity, diet, the use of glucocorticoids and so forth have to be taken into account [3]. Among them, genetic factors are found to play a pivotal role in the occurrence of osteoporosis and have received highly attention [4]. Large-scale twin studies observed that the heritability of bone loss among postmenopausal women was up to 56% [5, 6]. Many genes with innumerous gene polymorphisms have been identified to be covert risk alleles for osteoporosis vulnerability. Up to now, a series of candidate genes like vitamin D receptor [7, 8], estrogen receptors [9, 10], osteoprotegerin [11, 12], have been widely reported to be associated with osteoporosis.

Insulin-like growth factor-1 (IGF-1) is a single-chain polypeptide encoded by IGF-1 gene. IGF-1 is an important anabolic hormone and has the ability to regulate bone homeostasis and maintain skeletal architecture throughout adult life [13]. Literature reported that decreased serum concentration of IGF-1 strongly contribute to the occurrence of osteoporotic fractures in postmenopausal women [14]. Considering the vitally functional significance of the IGF-1, variations in the IGF-1 gene are possible susceptibility candidate loci for osteoporosis. In fact, Lee et al. had initially reported the relationship between IGF-1 gene polymorphisms and the BMD among postmenopausal women in 2002 [15]. Soon afterwards, a large sample studies led by Rivadeneira et al. [16] suggested that the microsatellite repeat polymorphism in the promoter of IGF-1 was linked to the BMD level and bone loss in postmenopausal women.

The single nucleotide polymorphisms (SNPs) located in IGF-1 gene also had been identified to be significantly associated with osteoporosis by Li and coworkers [17]. Subsequent replication studies tried to validate the association, but yielded conflicting results. Compared to a single gene-disease association study, a meta-analysis can strengthen the statistical power by combining individual study. A previous meta-analysis including four observational studies by Chen et al. [18] has been carried out. Considering the emergence of novel evidence on the association of IGF-1 polymorphisms and risk of osteoporosis, we set out to perform the present systematic review and meta-analysis to obtain a more credible association between IGF-1 polymorphisms and osteoporosis predisposition.

## Methods

This systematic review and meta-analysis conformed to the Preferred Reporting Items for Systematic Review and Meta-Analyses (PRISMA) guidelines [19].

### Literature search strategy

Five electronic databases including PubMed, EMBASE, ISI Web of Science, National Knowledge Infrastructure (CNKI), and Wanfang were methodically searched to identify genetic association studies involving the polymorphisms in IGF-1 gene published before November, 2017. For English databases, the search string was set using the combinations of Medical Subject Headings (MeSH) and free words as below: (Single Nucleotide Polymorphism or polymorphism or SNP or SNPs or “Polymorphism, Single Nucleotide”[Mesh]) and (“Insulin-Like Growth Factor I”[Mesh] or Somatomedin C or insulin-like growth factor 1 or IGF-1) and (“osteoporosis”[Mesh] or osteoporo* or bone loss or bone or fracture or “Fractures, Bone”[Mesh] or bone density or “Bone Density”[Mesh] or bone mineral density). For Chinese databanks, we adopt the following key words: “IGF-1|”| and |“duo tai xing” and “gu zhi shu song”. To prevent incomplete retrieval, the reference lists of related reviews and primary articles were manually searched to identify all potential studies. Two investigators (SG and ZL) accomplished the retrieving task independently.

### Inclusion and exclusion criteria

Studies included in our meta-analysis had to satisfy all of the following criteria: (1) osteoporosis diagnosed on the basis of radiological examination; (2) published studies aimed to assess the correlation between IGF-1 gene polymorphisms and osteoporosis; (3) case-control designed studies; (4) sufficient data provided for the calculation of the crude odds ratios (ORs) and 95% confidence intervals (95% CIs). Correspondingly, studies were excluded from our analysis for the following reasons: (1) case report, comments, reviews, or animal studies; (2) family-based studies; (3) data overlapping with previous article; (4) studies with unavailable alleles as well as genotype frequencies. For any discrepancy during this process, a consensus was achieved after discussion.

### Methodological quality assessment

The methodological quality of included studies was evaluated separately by two reviewers (SG and ZL) under the Newcastle-Ottawa Scale (NOS) for observational studies [20]. A ‘star system’ was applied to judge individual study on three broad perspectives: the selection of the case and control groups; the comparability of the case and control groups, and the ascertainment of either the exposure or outcome of interest for case-control studies (http://www.ohri.ca/programs/clinical_epidemiology/oxford.asp). Disagreements between two investigators were settled by discussion until consensus was reached.

### Data extraction

In compliance with the predetermined selection criteria, the following information was meticulously extracted by two reviewers (SG and ZL) from all qualified articles independently: (1) surname of the first author; (2) year of publication; (3) country where the study was conducted; (4) ethnicity of enrolled subjects; (5) numbers of case and control participants; (6) gender of the enrolled patients; (7) diagnostic criteria of osteoporosis; (8) genotypes distribution of case and control participants; (9) Hardy-Weinberg Equilibrium (HWE) test results of control subjects. In case of any discrepancy during this process, two authors re-inspected the article together and reached an agreement by mutual discussion.

### Statistical analysis

HWE for the control participants of each included study was evaluated by Chi-square test to assess goodness of fit. The strength of association between polymorphism in IGF-1 gene and risk of osteoporosis was presented as crude ORs accompanied by the corresponding 95% CI. To prevent an inflated false positive error rate, we did not conduct any assumption about the genetic model of inherence beforehand. The most appropriate genetic model was determined by a model-free approach [21]. If A variant was the gene of interest that could possibly lead to an increased or decreased risk of osteoporosis. OR1, OR2 and OR3 were calculated for genotypes AA versus aa, Aa versus aa, AA versus Aa for each polymorphism to capture the magnitude of genetic effect and to decipher the most plausible genetic model. Then the most reasonable genetic model of inherence was ascertained according to the associations between the three pairwise comparisons as follow:

1. Recessive model: if OR1 = OR3 ≠ 1 and OR2 = 1;
2. Dominant model: if OR1 = OR2 ≠ 1 and OR3 = 1;
3. Complete over-dominant model: if OR1 = 1, OR2 = 1/OR3 ≠ 1;
4. Co-dominant model: if OR1 > OR2 > 1 and OR1 > OR3 > 1, or OR1 < OR2 < 1 and OR1 < OR3 < 1.

After the underlying genetic model was verified, the counts of genotypes were collapsed into two categories to obtain the merged results. To evaluate the between-study heterogeneity, Q-statistical test and I^2^ test were employed. The random-effect model and fixed-effect model were utilized for data combination in the presence (*P* < 0.1, I^2^ > 50%) or absence of heterogeneity (*P* > 0.1, I^2^ < 50% indicates an acceptable heterogeneity) respectively [22, 23]. In the event of any statistically significant heterogeneity across studies, subgroup-analysis by source of the patients (both gender subjects or only post-menopausal women) were performed to look for the potential source of heterogeneity. The Leave-one-out sensitivity analysis was conducted by removing individual study one by one and reassessing the resulting effect on the estimate of overall effect. Egger’s regression test and Begg’s rank correlation test were exploited to estimate the publication bias (Stata version 12.0, Stata Corp LP, USA) [24]. In case that the number of included studies was smaller than ten, Trim and Fill method was also used to evaluate potential publication bias. Forest plots and funnel plot were originated from RevMan 5.3 software (Copenhagen: The Nordic Cochrane Centre, The Cochrane Collaboration, 2014).

## Results

### Literature search

**Fig. 1:**
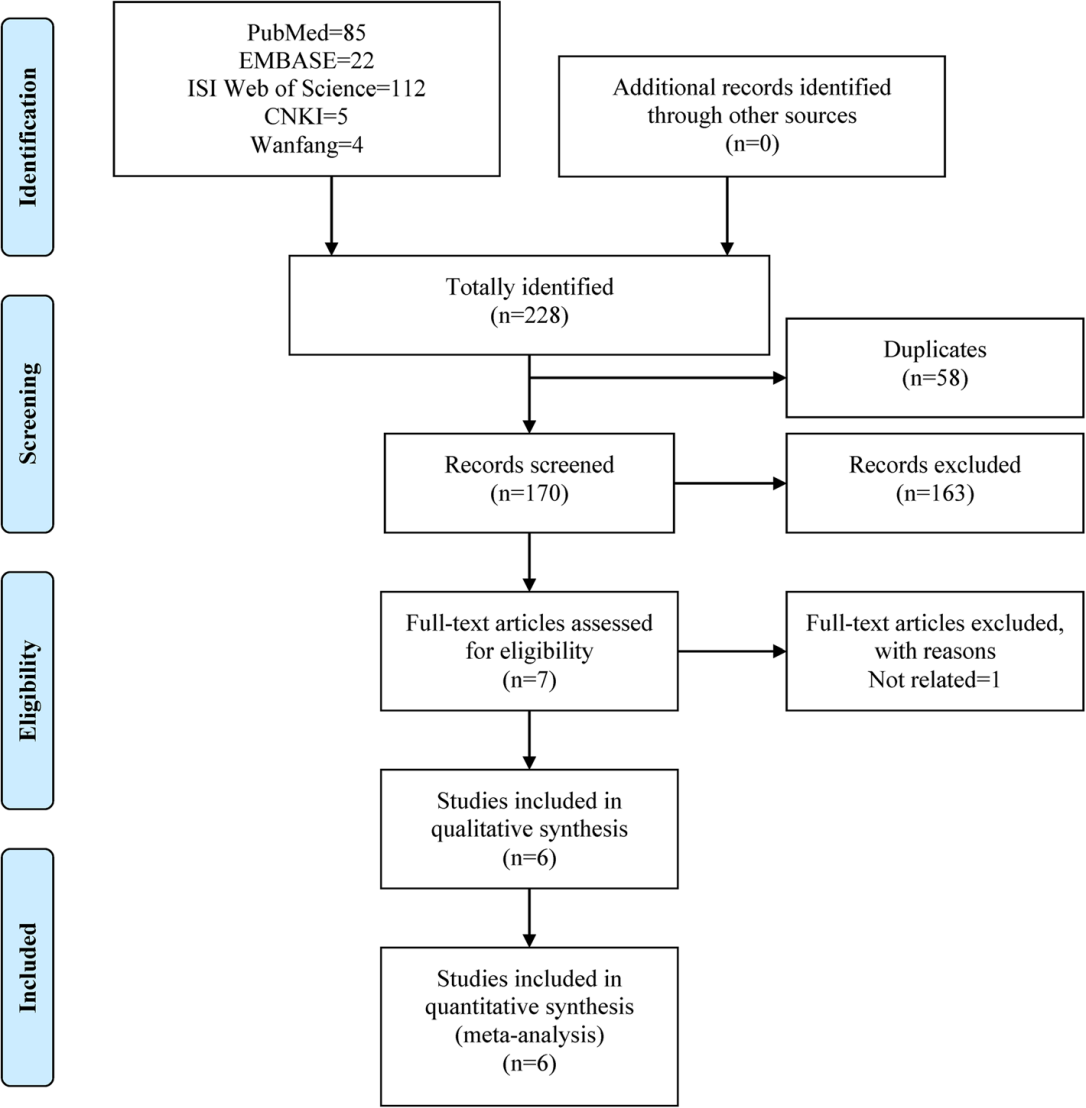
Flow chart of literature search and screen

Five online databases were hunted for potentially relevant studies and the initial literature. Combining the search results, a total of 228 records were yielded comprising 85 from PubMed, 22 from EMBASE, 112 from ISI Web of Science, 5 from CNKI and 4 from Wanfang, and no additional records were gained by the manual retrieval. After the first scanning, 58 duplicated records were rejected. Of the 170 potentially relevant remainders, a further 163 obviously improper articles were erased after reading the titles and abstracts. Among the remaining 7 records for full-text assessment, 1 unrelated was eliminated according to the predetermined inclusion and exclusion criteria. Ultimately, 6 articles [17, 25–29] were incorporated into the systematic review and meta-analysis. The literature selection process was presented in Fig. 1.

### Main characteristics

The main characteristics of included studies were shown in Table 1. Six case-control studies containing 2068 osteoporosis patients and 2071 healthy controls were identified and included in our meta-analysis. All the included studies were implemented across Chinese population, the sample-size ranged from 436 to 936. Of the six studies, five [17, 25–27, 29] referred to rs35767, rs2288377, and rs5742612. While Wei et al.’s study [28] focused on rs35767 and rs972936. Three studies [25, 27, 29] reported both male and female participants, yet the remaining three studies [17, 26, 28] focused on the association in only post-menopausal women subjects. The genotype frequencies of both case and control groups were summarized in Table 1. Six studies all conformed to the HWE in terms of rs35767. However, the majority of the studies did not comply with HWE when it came to rs2288377 and rs5742612, thus we did not perform meta-analyses for these two loci in the following section. On the basis of the NOS, all the six studies obtained an average of 6.3 stars for methodological quality assessment (Table 2).

**Table 1:** Main characteristics of included studies

Study	Country	Ethnicity	Sample size	Source of patients	Diagnostic criteria	Case			Control			HWE
*rs35767*						CC	CT	TT	CC	CT	TT	
Fan, 2017	China	Chinese	346/346	Both gender	WHO	154	163	29	152	157	37	0.932
Li, 2014	China	Chinese	216/220	PMOP women	WHO	95	94	27	114	89	17	0.998
Li, 2015	China	Chinese	485/485	PMOP women	WHO	202	210	74	238	201	46	0.932
Liu, 2017	China	Chinese	320/320	Both gender	WHO	152	136	32	156	131	33	0.781
Wei, 2015	China	Chinese	272/272	PMOP women	WHO	124	118	30	132	116	24	0.979
Zhang, 2015	China	Chinese	428/428	Both gender	WHO	182	193	53	216	186	26	0.233
*rs2288377*						AA	AT	TT	AA	AT	TT	
Fan, 2017	China	Chinese	346/346	Both gender	WHO	118	171	57	164	160	22	0.113
Li, 2014	China	Chinese	216/220	PMOP women	WHO	182	21	13	189	21	10	**< 0.001**
Li, 2015	China	Chinese	485/485	PMOP women	WHO	396	52	36	413	45	27	**< 0.001**
Liu, 2017	China	Chinese	320/320	Both gender	WHO	125	145	50	157	138	25	0.781
Zhang, 2015	China	Chinese	428/428	Both gender	WHO	349	44	35	365	40	23	**< 0.001**
*rs5742612*												
Fan, 2017	China	Chinese	346/346	Both gender	WHO	298	30	18	301	35	10	**< 0.001**
Li, 2014	China	Chinese	216/220	PMOP women	WHO	178	21	17	183	21	16	**< 0.001**
Li, 2015	China	Chinese	485/485	PMOP women	WHO	389	55	41	400	50	35	**< 0.001**
Liu, 2017	China	Chinese	320/320	Both gender	WHO	264	33	23	245	69	6	0.907
Zhang, 2015	China	Chinese	428/428	Both gender	WHO	336	48	44	346	42	41	**< 0.001**

PMOP: post-menopausal; WHO: World Health Organization criteria; HWE: Hardy-Weinberg Equilibrium

Statistically significant findings appeared in bold

**Table 2:** Methodological quality of included studies

Item/Study	Fan, 2017	Li, 2014	Li, 2015	Liu, 2017	Wei, 2015	O’Connell, 2014
Adequate definition of cases	*	*	*	*	*	*
Representativeness of cases	–	–	–	–	*	–
Selection of control subjects	*	–	–	–	–	–
Definition of control subjects	*	*	*	*	*	*
Control for important factor or additional factor	*	*	**	–	**	*
Exposure assessment	*	*	*	*	*	*
Same method of ascertainment for all subjects	*	*	*	*	*	*
Non-response rate	*	*	*	*	*	*

A study could be awarded a maximum of one star for each item except for the item “Control for important factor or additional factor”

The definition/explanation of each column of the Newcastle-Ottawa Scale is available from http://www.ohri.ca/programs/clinical_epidemiology/oxford.asp

### Meta-analyses and subgroup-analyses

**Fig. 2:**
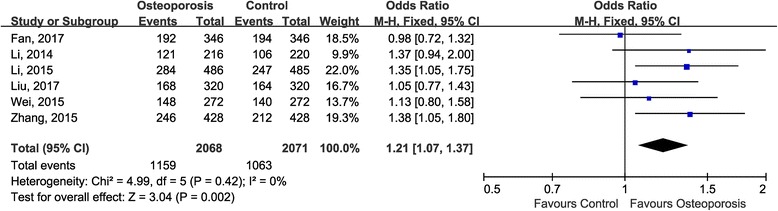
Forest plot of association between rs35767 and risk of osteoporosis using the dominant model

Previous to pooling data of all the included studies, we carried out testable hypotheses to look for the best-matching genetic model of inherence. The estimated OR1 (TT/CC: 1.45, 95% CI 1.01, 2.07; *P* = 0.04) and OR2 (TC/CC: 1.15, 95% CI 1.01, 1.31; *P* = 0.03) were both statistically significant whereas the estimated OR3 (TT/TC: 1.27, 95% CI 0.95, 1.69; *P* = 0.11) was insignificant, indicating that the dominant model was the most reasonable genetic model for meta-analysis. When using the dominant model, the counts of genotypes of TT and TC groups were combined and compared with CC groups. Since no between-study heterogeneity across the included studies was detected (*P* = 0.42, I^2^ = 0), the fixed-effect model was adopted for statistical analysis. The summarized OR suggested that there existed a statistically significant association between rs35767 in IGF-1 gene and the osteoporosis predisposition (OR 1.21, 95% CI 1.07, 1.37; *P* = 0.002) (Fig. 2).

**Fig. 3:**
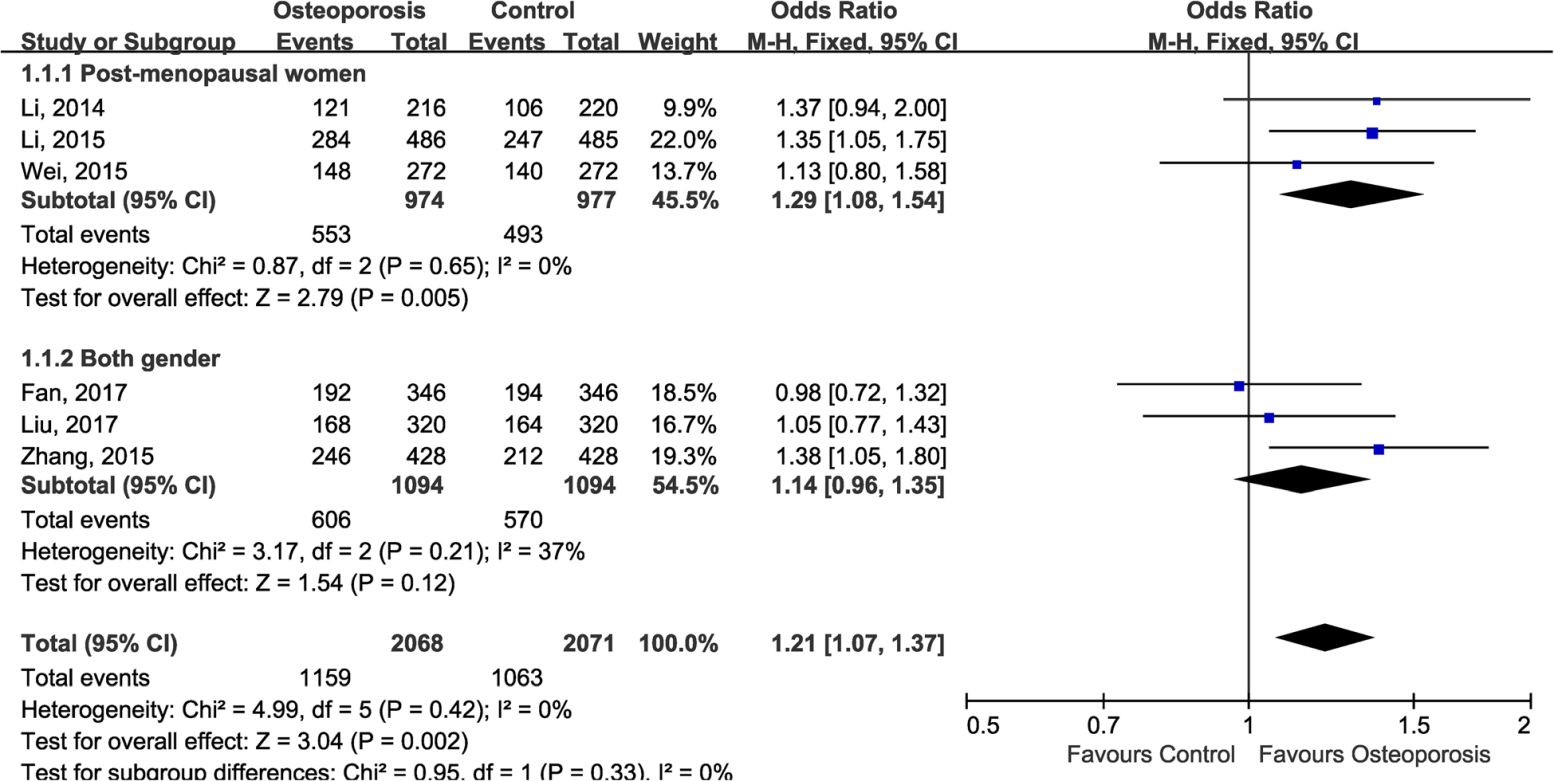
Subgroup-analysis by source of patients

Even though no heterogeneity was observed, we performed subgroup-analysis according to source of the patients to evaluate the association between rs35767 and osteoporosis susceptibility in both subgroups (Fig. 3). The results suggested that rs35767 was significantly correlated to the risk of osteoporosis in post-menopausal subgroup (OR 1.29, 95% CI 1.08, 1.54; *P* = 0.005), but the association was not established in the both gender subgroup (OR 1.14, 95% CI 0.96, 1.35; *P* = 0.12).

### Sensitivity analysis and publication bias

**Fig. 4:**
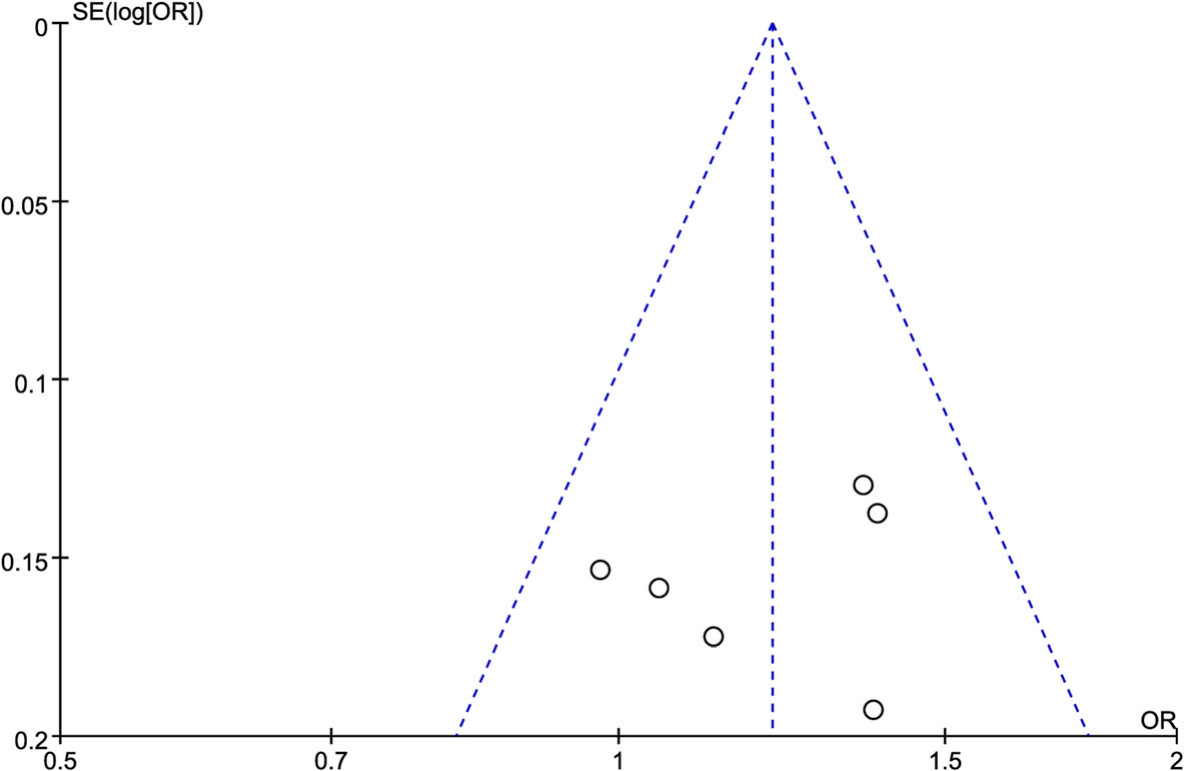
Funnel plot of association between rs35767 and risk of osteoporosis

The sensitivity analysis was performed by sequential excluding each of the eligible studies. While the corresponding pooled OR appeared to be not significantly affected, indicating a robust and stable result (Additional file 1: Figure S1). The funnel plot was visually symmetrical (Fig. 4), the Egger’s test (*t* = − 0.64, *P* = 0.555) and Begg’s test (z = 0.00, *P* = 1.000) also suggested no statistically significant publication bias. Trim and Fill method showed that the association between rs35767 polymorphism and risk of osteoporosis was stable (number to trim = 0, data were unchanged).

## Discussion

With the general aging of societies, osteoporosis is growing into a serious health threat and has given rise to significant physical, psychosocial, and financial consequences to the sufferers. Given genetic factors play a leading role in the histogenesis and development of osteoporosis, identification of polymorphisms in potential pathogenicity genes enable us to predict the disease and take preventive measures, as well as determine potential targets for specific pharmacologic treatment. Association studies in human have defined a range of polymorphisms in several genes which regulate BMD and osteoporosis [30]. Among them, IGF-1 is one of the most commonly studied candidate genes due to its critical role in the regulation of bone turnover and homeostasis.

IGF-I is a ubiquitous hormone that expressed in virtually every tissue, but the circulating IGF-1 is mainly produced and secreted in the liver tissue via the regulation of growth hormone [31].The mature IGF-1 polypeptide is encoded by exons 3 and 4 of IGF-1 gene, which comprises six exons [13]. Both the systemic and local skeletal IGF-1 act as a central growth factor in the regulation of body formation and the maintenance of bone mass by enhancing bone matrix formation as well as inhibiting bone degradation [13, 32]. It was estimated that more than 40% of basal bone cell proliferation could be blocked by inhibiting the actions of IGF-1 produced endogenously by bone cells in vitro study [33]. During the individual development and maturity, IGF-1 plays a key role in regulating longitudinal skeletal growth. Apart from this effect, IGF-1is much better known as an anabolic hormone which has the ability to determine bone modeling and remodeling throughout life. The anabolic effect of IGF-1 was mainly exerted on cortical and trabecular bone, and it is also of great importance for the achievement of the peak bone mass, which is a critical contributing factor for future risk of osteoporosis [34]. IGF-1 levels decrease with advancing age, Liu et al. [35] found the serum IGF-1 level started to decline at age 30, nearly 20 years earlier than the significant loss of BMD, thus making IGF-1 as a predictor for early bone loss. A large body of literature have also reported that IGF-1 and several IGF-binding proteins (IGFBPs), like IGFBP-1, 3, 4, and 5, were positively correlated with bone mass and could serve as independent predictors for the occurrence of osteoporosis and fracture [36].

There have been a series of studies looking into the association between the rs35767 polymorphism in IGF-1 gene and osteoporosis predisposition, while these results acquired were conflicting and as yet no robust evidence was available on this association. With limited sample size of individual study, it is quite difficult to draw a convincing conclusion because of low statistical validity. To overcome this drawback, the systematic review and meta-analysis was a capable alternative [37]. The present study focused on the elderly Chinese population. In the summarized analysis of 2068 osteoporosis cases and 2071 healthy controls, rs35767 polymorphism was investigated to be significantly correlated to osteoporosis susceptibility. To be specific, the TT + TC genotype appeared to increase the risk of osteoporosis. However, in the aspect of subgroup-analysis based on gender stratification, only the postmenopausal individuals carrying the TT + TC genotype had a significantly higher osteoporosis vulnerability, which sustained the supposition that postmenopausal women were at a higher risk for osteoporosis.

It should be pointed out that Chen et al. [18] recently published a paper that shared a similar result with our present one. But our study has several methodological advantages. Firstly, we established a more comprehensive literature search strategy for potentially eligible literature, and the literature search and screen yielded six studies including 2068 cases and 2071 controls, while Chen et al. [18] only obtained four studies with 1402 cases and 1405 controls. Except for four studies [17, 26, 28, 29] included by Chen et al., we included two additional recently published studies [25, 27] to increase the statistical power of our study. Secondly, Chen et al. [18] performed their meta-analysis using multiple genetic models of inheritance without applying a correction for multiple testing. False-positive results may arise through a failure to correct for multiple testing. In contrast, we employed a model-free approach to avoid this issue. Thirdly, our meta-analysis appraised the HWE of control participants of each included study which also contributed to the credibility of our results.

Although the present meta-analysis has provided a more comprehensive evaluation and precise evidence of the currently available data on the association between the IGF-1 gene polymorphism and osteoporosis, there are still several limitations that should be pointed out. First of all, all the included studies were implemented in Chinese population with limited sample sizes, the result could not be generalized into the whole population. In consideration of the possible ethnic differences, deeper investigations on various populations are warranted to validate the current result. Besides, we only investigated the role of individual locus polymorphism in IGF-1gene separately, but more than one of the loci might interplay with each other and function together to advance osteoporosis. In addition, as with other complex disorders, osteoporosis risk was modulated by multiple genetic factors with synergetic effect other than IGF-1 gene. Therefore, an investigation of the combined interaction of these related genes is necessary to completely elucidate the pathogenesis of osteoporosis. Lastly, only articles in English and Chinese from five databases were retrieved for our meta-analysis, potential relevant articles published in other languages might have been skipped, which might incur inclusion criteria bias [38].

## Conclusion

The findings of our current study suggested a significant association between rs35767 polymorphism and risk of osteoporosis in Chinese postmenopausal women. To confirm the definite gene-disease association between IGF-1 polymorphisms and susceptibility of osteoporosis, additional studies within other ethnic backgrounds are strongly encouraged.

## Additional files

Additional file 1: **Figure S1**. Forest plot of sensitivity-analysis, given named study is omitted. The sensitivity analysis was performed by sequential excluding each of the eligible studies. While the corresponding pooled odds ratio (OR) appeared to be not significantly affected, indicating a robust and stable result.(TIF 1695 kb).

## Abbreviations

BMD: Bone mineral density
CNKI: China National Knowledge Infrastructure
HWE: Hardy-Weinberg equilibrium
IGF-1: Insulin-like growth factor-1
IGFBP: IGF-binding protein.
MeSH: Medical Subject Headings
NOS: Newcastle-Ottawa Scale
OR: Odds ratio; 95% CI, 95% confidence interval
PRISMA: Preferred Reporting Items for Systematic Review and Meta-Analyses
SNP: Single nucleotide polymorphism
