# Supplementary figures and images for: Association between IGF-1 polymorphisms and risk of osteoporosis in Chinese population: a meta-analysis
Source: BMC Musculoskelet Disord. 2018 May 10;19:141. doi: 10.1186/s12891-018-2066-y (PMC5944070; doi:10.1186/s12891-018-2066-y)

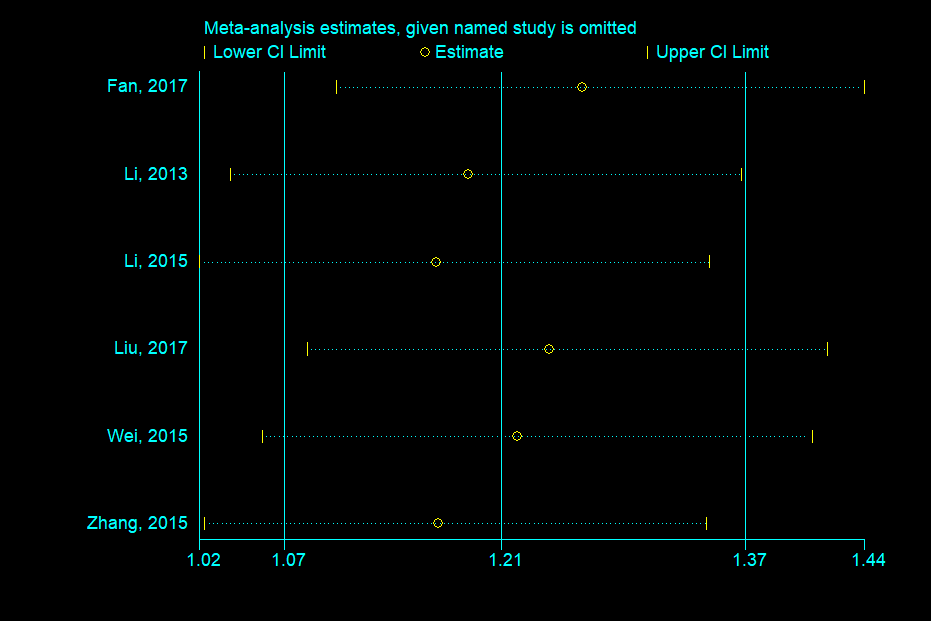

Supplement: Supplementary file 1 — Figure S1. Forest plot of sensitivity-analysis, given named study is omitted. The sensitivity analysis was performed by sequential excluding each of the eligible studies. While the corresponding pooled odds ratio (OR) appeared to be not significantly affected, indicating a robust and stable result.(TIF 1695 kb). [file 12891_2018_2066_MOESM1_ESM.tif]
